# Protective Effects of *Phellinus linteus* Mycelium on the Development of Osteoarthritis after Monosodium Iodoacetate Injection

**DOI:** 10.1155/2020/7240858

**Published:** 2020-08-15

**Authors:** Mi-Rae Shin, Jin A Lee, Min Ju Kim, Hae-Jin Park, Byeong Wook Park, Seung Bo Seo, Seong-Soo Roh

**Affiliations:** ^1^Department of Herbology, Korean Medicine of College, Daegu Haany University, 136, Shinchendong–ro, Suseong-gu, Deagu 42158, Republic of Korea; ^2^DHU Bio Convergence Testing Center, 1, Hanuidae-ro, Gyeongsan-si, Gyeongsangbuk-do 38610, Republic of Korea; ^3^Hankook Shinyak Pharm. Co., Ltd., 39-83 Zhongshan-gil, Yangchon-myeon, Nonsan-si, Chungcheongnam-do 33023, Republic of Korea

## Abstract

**Objective:**

The aim of this study was to identify the protective effects of *Phellinus linteus* mycelium (PLM) and its possible mechanisms in a model of monosodium iodoacetate- (MIA-) induced osteoarthritis (OA).

**Methods:**

Intra-articular injection of MIA was injected to 50 *μ*L with 80 mg/mL using a 0.3 mL insulin syringe into the right knee joint. Changes in hindpaw weight-bearing distribution between the right (osteoarthritic) and left (contralateral control) legs were used as an index of joint discomfort. PLM (50, 100, and 200 mg/kg body weight) was orally administered once daily for 14 days from day 7 after MIA treatment. And then, various factors associated with inflammatory response and cartilage degeneration in cartilage tissues detected by western blotting.

**Results:**

PLM treatment showed a concentration-dependent elevation in change in hindpaw weight-bearing distribution (HWBD). PLM200 demonstrated the capacity to significantly increase HWBD, indicating that the change in weight-bearing distribution means the reduction of spontaneous pain. Our results indicate that PLM suppressed the inflammatory factors via NF-*κ*B signaling pathway induced by p38 phosporlyation. Moreover, PLM200 exhibited a significant reduction of ROS produced by the nicotinamide adenine dinucleotide phosphate (NADPH) oxidase. PLM100 and PLM200 inhibited the levels of matrix metalloproteinase (MMP)-1, one of proteinase that degrades extracellular matrix (ECM).

**Conclusions:**

Taken together, our results indicated that PLM has a strong chondroprotective effect through the suppression both ROS production and inflammation.

## 1. Introduction

Osteoarthritis (OA) is nowadays one of the most prevalent chronic arthritis of human joint, and age and obesity are well known for its powerful risk factors [1, 2]. OA is characterized by cartilage degradation and inflammation and develops progressively over several years. Consequently, OA causes physical limitations, disability, mental stress, and socioeconomic burden [3]. Therefore, both bodily pain and physical functioning by OA are lower than that for cardiovascular conditions, chronic respiratiory diseases, and gastrointestinal conditions [4]. OA leads to much lower quality of life. Although OA was referred to as the noninflammatory arthritis historically, it is considered a low-grade inflammation disease [5, 6] triggered by factors like biomechanical stress associated with OA development [7]. Therefore, anti-inflammatory drugs like nonsteroidal anti-inflammatory drugs (NSAIDs) were given to patients with symptomatic OA to remove OA symptoms [8, 9]. However, these drugs led the gastrointestinal and cardiovascular side-effects [10]. Accordingly, new strategies about an effective and a safe herbal medicine for OA treatment is urgently needed.

Currently, many studies focused on describing the key factors responsible for the deepening of inflammatory processes [11]. An analysis of the ever-increasing reports showed the important role of the cytokine network in the pathogenesis of OA [12]. During the progression of OA, the degree of cytokine production can make changes to the duration and severity of OA [13]. Such inflammatory cytokines including tumor necrosis factor (TNF)-*α*, interleukin (IL)-1*β*, and IL-6 are important factors participating in the pathogenesis of OA [14] and intimately related to cartilage matrix degradation. Matrix metalloproteinases (MMPs), proteolytic enzymes, are a family of zinc-dependent endoproteases and possess multiple roles in degradation of various proteins and tissue remodeling in the extracellular matrix (ECM). Moreover, MMPs promote cell migration, proliferation, and differentiation and could play a role in cell apoptosis, tissue repair, and angiogenesis [15]. In addition, MMPs expression also increases during the inflammatory process. MMPs are secreted by inflammatory cells and promoted by inflammatory cytokines [16]. Whereas, it generally counteracts inhibitory actions of endogenous TIMPs [17, 18]. Hence, the inhibition of inflammatory cytokines and MMPs may be a valid approach for OA treatment.

Medicinal fungi could produce diverse bioactive metabolites including anticancer drugs, antibiotics, and immunosuppressants [19–21]. Many medically meaningful metabolites had been reported from various edible fungi. *Phellinus linteus* is a famous medicinal mushroom that is widely used in Korea, Japan, China, and other Asian countries [22]. The various components including polyphenols, pyrans, polysaccharides, and triterpenoids play a significant role in improving the health condition [23, 24]. Above all, *Phellinus linteus* mycelium (PLM) was used as medicines or healthy foods to treat several diseases such as infections, ulcer, cancer, and diabetes [25]. In the previous reports, PLM showed wide spectrum of bioactivities, such as antioxidant, anti-inflammatory, cytotoxic, antiviral, and antidiabetic effects [26–28]. Especially, *β*-glucan isolated from mushroom is a natural polysaccharide and possess various biological effects such as immune-modulating properties, antitumor, anti-infection, and lowering blood cholesterol [29, 30].

The hypothesis of the current study was PLM supplementation could have chondroprotective effect via the inhibition of cartilage matrix degradatin on the articular cartilage. So, the efficacy and underlying mechanism of PLM were examined using the MIA-induced OA model.

## 2. Materials and Methods

### 2.1. Materials

Monosodium iodoacetate (MIA), phenyl methyl sulfonyl fluoride (PMSF), dithiothreitol (DTT), and diethylenetriaminepentaacetic acid (DTPA) were purchased from Sigma Aldrich Co., Ltd (St. Louis, MO, USA). The protease inhibitor mixture solution and ethylene diamine tetraacetic acid (EDTA) were purchased from Wako Pure Chemical Industries, Ltd. (Osaka, Japan). ECL western blotting detection reagents and pure nitrocellulose membranes were supplied by GE Healthcare (Piscataway, NJ, USA). 0.3 mL insulin syringe was obtained from BD Medical-Diabetes Care (Holdrege, USA). Besides, all other chemicals and reagents were purchased from Sigma Aldrich Co., Ltd. (St Louis, MO, USA). Primary antibody rabbit polyclonal antibodies against p47^phox^, p22^phox^, catalase, glutathione peroxidase-1/2 (GPx-1/2), phospho-p38 (p-p38), goat polyclonal antibodies against tumor necrosis factor alpha (TNF-*α*), interleukin-6 (IL-6), interleukin-1 beta (IL-1*β*), mouse monoclonal antibodies against nuclear factor-kappa B p65 (NF-kBp65), cyclooxygenase 2 (COX-2), inducible nitric oxide synthase (iNOS), matrix metalloproteinase-1 (MMP-1), tissue inhibitor matrix metalloproteinase 1 (TIMP-1), histone, *β*-actin, and secondary antibody were obtained from Santa Cruz Biotechnology (Santa Cruz, CA, USA). Moreover, rabbit polyclonal antireduced nicotinamide adenine dinucleotide phosphate oxidase 4 (NOX4) was obtained from LifeSpan BioSciences (Seattle, WA, USA). The BCA protein assay kit is supplied by Thermo Fisher Scientific (Waltham, MA, USA).

### 2.2. Preparation of PLM

*Phellinus linteus* mycelium was obtained as a dried mycelium from Hankook Shinyak Corp. (Nonsan-si, Korea), and Green-lipped mussel powder was obtained from McFarlane Marketing (Aust) Pty Ltd. (Melbourne, Australia). *Phellinus linteus* mycelium was inoculated under optimal culture condition (at 30°C and pH 4-5) for 14 days on yeast malt extract glucose (YMG) agar. The extract condition was optimized for extraction temperature (100°C), extraction time (24 h), solvent amount (10 times purified water), and extraction frequency (1st). A 50 ton large-scale incubator was used to produce 7 g/L dry mycelium in 4 days after mass production of mushroom mycelium through the following manufacturing process (Table 1). The produced PLM analyzed the nutritional components in accordance with the method of food revolution at Korea Health Supplement Institute (Seongnam, Korea) [31]. The composition of PLM is shown in Table 2.

### 2.3. *β*-Glucan Measurement of PLM

*β*-Glucan content in PLM was analysed using the K-YBGL kit (Megazyme, Ireland) as the mushroom and yeast *β*-glucan assay procedure. Extraction, laboratory analysis, and calculation were performed in accordance with the manufacturer's instructions. *β*-glucan content was calculated as a percentage using the following equation:

(1) $$\begin{array}{c} \beta -\text{Glucan content}\left(\%\right)=\left(\text{total glucan content}\right)-\left(\text{content excluding }\beta -\text{glucan}\right). \end{array}$$

Consequently, *β*-glucan content of PLM was 14.16 ± 6.27%.

### 2.4. Development of OA with MIA Injection and PLM Administration

Seven-week-old male Sprague Dawley rats weighing 200–250 g at the start of the experiment were purchased from DBL Co. (Eumseong, South Korea). The animals were housed three per cage in a room with controlled temperature conditions (23 ± 2°C), humidity (about 55 ± 5%), and lighting (12 h light/dark cycle) with free access to food and water. After adaptation (1 week), rats were randomly arranged in the descending order of weight and assigned into six groups of equal numbers (*n* = 7) without any statistical significance among the groups:

  Group 1: Nor group included the normal rats
  Group 2: Con group included the MIA control rats
  Group 3: GLM200 group included the green-lipped mussel 200 mg/kg-administered and MIA rats
  Group 4: PLM50 group included the PLM 50 mg/kg-administered and MIA rats
  Group 5: PLM100 group included the PLM 100 mg/kg-administered and MIA rats
  Group 6: PLM200 group included the PLM 200 mg/kg-administered and MIA rats

The normal and MIA control groups were administrated water using a stomach tube, while the other groups were orally administered green-lipped mussel 200 mg/kg or PLM 50, 100, and 200 mg/kg using a stomach tube for 2 weeks. OA was induced in SD rats; we employed the method of Wang with minor modifications [32]. After anesthetization with injection of Zoletil mixture (Vibrac, France) 0.75 mg/kg intraperitoneally, rats were injected with MIA 80 mg/mL in a 50 *μ*L volume using a 0.3 mL insulin syringe (31G needle) inserted through the patellar ligament into the intra-articular space of the right knee; normal rats were injected with an equivalent volume of saline [33]. The rats in all groups were sacrificed after the end of experimental period. The rats were anesthetized using a mixture of Zoletil and xylazine and euthanized by isoflurane overdose. Right knee joint specimens were collected, and the muscles around the knee were joint quickly removed. The femoral cartilage of rat was removed through circular incision along the femoral edge of the articular capsule. In addition, the femur and tibial fibula were cut off along each bone surface. And then, the joint capsule was cut longitudinally and the synovial tissue was separated. The cartilage specimens were stored at −80°C until further study. The terminal blood samples were centrifuged at 3000 rpm for 20 min at 4°C and the serum was collected and stored at −80°C until analysis.

### 2.5. Measurements of Hindpaw Weight-Bearing Distribution

Hindpaw weight-bearing distribution (HWBD) was assessed using an incapacitance meter that measures the distribution of weight bearing across each hindlimb. The balance in the weight-bearing capability of the hindpaws was disrupted after OA induction. A significant shift in weight from the arthritic site to the contralateral limb, i.e., a weight bearing deficit, was considered to be an index of pain. Pain was measured via the weight bearing of the paw load using an incapacitance tester (Linton Instrumentation, Norfolk, UK) [34]. The hinpaw weight bearing distribution ratio (%) was calculated using the following equation:

(2) $$\begin{array}{c} \left[\frac{\text{weight on right hind limb}}{\text{weight on left hind limb}+\text{weight on right hind limb}}\right]\times 100. \end{array}$$

### 2.6. Preparation of Cytosol and Nuclear Fractions

Protein extraction was performed according to the method of Komatsu with minor modifications [35]. The cytosol fraction was homogenized with ice-cold lysis buffer A (250 mL) containing 10 mM HEPES (pH 7.8), 10 mM KCl, 2 mM MgCl_2_, 1 mM DTT, 0.1 mM EDTA, 0.1 mM PMSF, and 1,250 *μ*L protease inhibitor mixture solution. The homogenate was incubated at 4°C for 20 min. And then, 10% NP-40 was added and mixed well. After centrifugation (13,400 ×g for 2 min at 4°C) using Eppendorf 5415R (Hamburg, Germany), the supernatant liquid (cytosol fraction) was separated into a new e-tube. The left pellets were washed twice by buffer A, and the supernatant was discarded. Next, the pellets were suspended with lysis buffer C (20 mL) containing 50 mM HEPES (pH 7.8), 50 mM KCl, 300 mM NaCl, 1 mM DTT, 0.1 mM EDTA, 0.1 mM PMSF, 1% (v/v) glycerol, and 100 *μ*L protease inhibitor mixture solution suspended and incubated at 4°C for 30 min. After centrifugation (13,400 ×g for 10 min at 4°C), the nuclear fraction was prepared to collect the supernatant. The protein concentration was determined using BCA protein assays by Thermo Fisher Scientific (Waltham, USA). Both cytosol and nuclear fractions were kept at −80°C before the analysis.

### 2.7. Immunoblotting Analysis

For the estimation of NF-*κ*Bp65 and histone (1 : 1000; Santa Cruz Biotechnology, Dallas, TX, USA), 10 *μ*g of proteins from each nuclear fraction was electrophoresed through 8–10% sodium dodecylsulfate polyacrylamide gel (SDS-PAGE). Separated proteins were transferred to a nitrocellulose membrane, blocked with 5% (w/v) skim milk solution for 1 h, then incubated with primary antibodies to NF-*κ*Bp65 and histone, respectively, overnight at 4°C. After the blots were washed, they were incubated with anti-mouse IgG HRP-conjugated secondary antibody (1 : 3000; Santa Cruz Biotechnology) for 1.5 h at room temperature. In addition, 10–12 *μ*g proteins of each cytosol fraction was eletrophoresed in 10–14% sodium dodecylsulfate polyacrylamide gel (SDS-PAGE) for immunodetection of p-p38/COX-2/iNOS/TNF-*α*/IL-6/IL-1*β*/catalase/GPx-1/2/MMP-1/TIMP-1/*β*-actin (1 : 1000; Santa Cruz, USA). Each antigen-antibody complex was visualized using ECL western blotting detection reagents and detected by chemiluminescence with Sensi-Q 2000 Chemidoc (Lugen Sci Co., Ltd., Gyeonggi-do, Korea). Band densities were measured using ATTO Densitograph software (ATTO Corporation, Tokyo, Japan) and quantified as the ratio to histone or *β*-actin. The protein levels of the groups are expressed relative to those of the normal rat (represented as 1). We followed the methods of Mi-rae Shin et al. 2017 [36].

### 2.8. Statistical Analysis

Data are expressed as mean ± SEM. Statistical comparisons were assessed by one-way ANOVA followed by the least-significant differences (LSD) test (SPSS 22.0 for Windows, SPSS Inc., NY, USA). Values of *P* < 0.05 were considered significant.

## 3. Results

### 3.1. Change in Hindpaw Weight-Bearing Distribution

**Figure 1:**
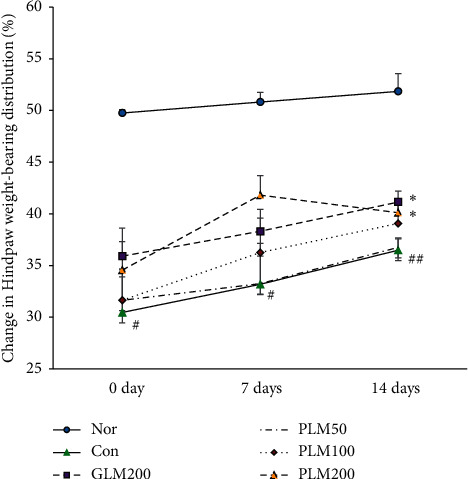
Change in Hindpaw weight-bearing distribution. Values are the mean ± SEM (*n* = 7). Nor: normal rats; Con: MIA-induced osteoarthritis rats; GLM200: MIA-induced osteoarthritis rats administrated with green-lipped mussel 200 mg/kg body weight; PLM50: MIA-induced osteoarthritis rats administrated with PLM 50 mg/kg body weight; PLM100: MIA-induced osteoarthritis rats administrated with PLM 100 mg/kg body weight; PLM200: MIA-induced osteoarthritis rats administrated with PLM 200 mg/kg body weight. Significance: ^#^*P* < 0.05, ^##^*P* < 0.01 vs. normal rat values. ∗After 2 weeks, the decreased HWBD was significantly increased at GLM200 and PLM200 groups (*P* < 0.05).

The MIA model is the most often used, being commonly chosen to evaluate the efficacy of pharmacological agents for pain management. Pain assessment assay commonly used is the incapacitance test, which measures the weight distribution between both hind limbs [37]. The pain of knee joint leads to decrease of hindpaw weight-bearing distribution (HWBD), which has been proved in the previous study [34]. The HWBD on 0, 1, and 2 weeks is measured in Figure 1. On day 0, the HWBD of MIA-treated groups was lower compared with that of the normal group (*P* < 0.05). After 2 weeks, the decreased HWBD was significantly increased at GLM200 and PLM200 groups (*P* < 0.05). The pain of knee joint could alleviate though PLM supplementation.

### 3.2. NOX4, p47^phox^, and p22^phox^ in Cartilage Tissues

**Figure 2:**
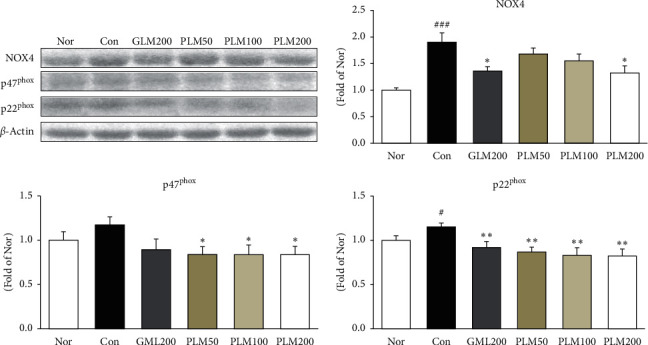
NADPH oxidases: NOX4, p47^phox^, and p22^phox^ in cartilage tissues. Values are the mean ± SEM (*n* = 6). Nor: normal rats; Con; MIA-induced osteoarthritis rats; GLM200: MIA-induced osteoarthritis rats administrated with green-lipped mussel 200 mg/kg body weight; PLM50: MIA-induced osteoarthritis rats administrated with PLM 50 mg/kg body weight; PLM100: MIA-induced osteoarthritis rats administrated with PLM 100 mg/kg body weight; PLM200: MIA-induced osteoarthritis rats administrated with PLM 200 mg/kg body weight. Significance: ^#^*P*< 0.05, ^###^*P* < 0.001 vs. normal rat values and ^∗^*P* < 0.05, ^∗∗^*P* < 0.01 vs. MIA control rat values.

The NADPH oxidase family including NOX4, p47^phox^, and p22^phox^ is one of the potent cellular sources in the overproduction of reactive oxygen species (ROS) [38]. The expressions of NOX4, p47^phox^, and p22^phox^ were examined, as shown in Figure 2. The treatment of MIA significantly led to upregulation of both NOX4 and p22^phox^ in the cartilage tissue; however, PLM200 administration significantly suppressed the levels of all markers (NOX4; *P* < 0.05, p47^phox^; *P* < 0.05, and p22^phox^; *P* < 0.01).

### 3.3. p38-MAPK and NF-*κ*Bp65 in Cartilage Tissues

**Figure 3:**
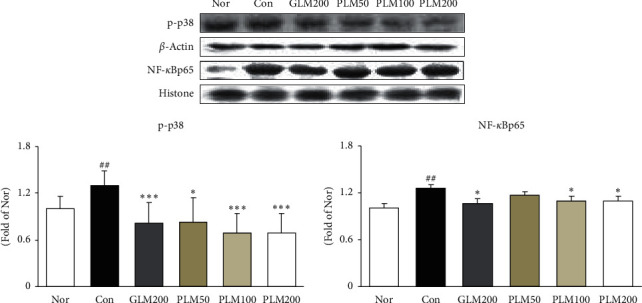
p-p38 and NF-*κ*Bp65 protein expressions in cartilage tissues. Values are the mean ± SEM (*n* = 6). Nor: normal rats; Con: MIA-induced osteoarthritis rats; GLM200: MIA-induced osteoarthritis rats administrated with green-lipped mussel 200 mg/kg body weight; PLM50: MIA-induced osteoarthritis rats administrated with PLM 50 mg/kg body weight; PLM100: MIA-induced osteoarthritis rats administrated with PLM 100 mg/kg body weight; PLM200: MIA-induced osteoarthritis rats administrated with PLM 200 mg/kg body weight. Significance: ^##^*P* < 0.01 vs. normal rat values and ^∗^*P* < 0.05, ^∗∗∗^*P* < 0.001 vs. MIA control rat values.

The previous study reported that p38-MAPK activity attenuates the transcriptional activity of the proinflammatory transcription factor, NF-*κ*B [39, 40]. Activation of p38-MAPK affected the NF-*κ*Bp65 protein expression (Figure 3). The injection of MIA resulted in a significant increase of p38-MAPK compared with the normal group. In contrast, the treatment of all drugs significantly reduced the expression of p38-MAPK. Besides that, the NF-*κ*Bp65 expression in MIA control was elevated, whereas it was attenuated by GLM200, PLM100, and PLM200 administration (*P* < 0.05).

### 3.4. TNF-*α*, IL-6, IL-1*β*, COX-2, and iNOS in Cartilage Tissues

**Figure 4:**
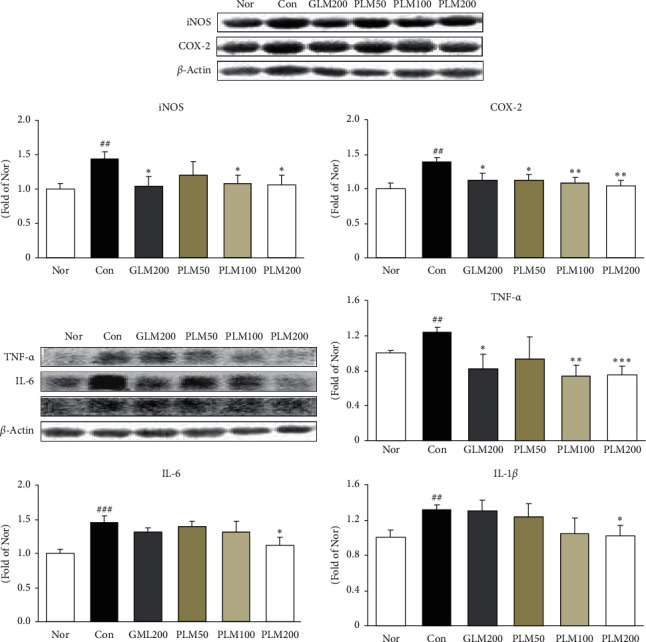
iNOS, and COX-2, TNF-*α*, IL-6, and IL-1*β* protein expressions in cartilage tissues. Values are the mean ± SEM (*n* = 6). Nor: normal rats; Con: MIA-induced osteoarthritis rats; GLM200: MIA-induced osteoarthritis rats administrated with green-lipped mussel 200 mg/kg body weight; PLM50: MIA-induced osteoarthritis rats administrated with PLM 50 mg/kg body weight; PLM100: MIA-induced osteoarthritis rats administrated with PLM 100 mg/kg body weight; PLM200: MIA-induced osteoarthritis rats administrated with PLM 200 mg/kg body weight. Significance: ^##^*P* < 0.01: ^###^*P* < 0.001 vs. normal rat values and ^∗^*P* < 0.05: ^∗∗^*P* < 0.01: ^∗∗∗^*P* < 0.001 vs. MIA control rat values.

Activated NF-*κ*B regulates the expression of inflammatory mediators and many cytokines [14].  Figure 4 displays the effect of PLM on the expression of inflammation-related mediators such as COX-2, iNOS, and cytokines including TNF-*α*, IL-6, and IL-1*β*. The MIA treatment significantly augmented these protein expressions. On the other hand, PLM200 treatment significantly decreased all factors. Especially, the suppression effects of PLM200 was near the levels of the normal group (iNOS, IL-6, and IL-1*β*; *P* < 0.05, COX-2; *P* < 0.01, TNF-*α*; *P* < 0.001).

### 3.5. MMP-1 and TIMP-1 in Cartilage Tissues

**Figure 5:**
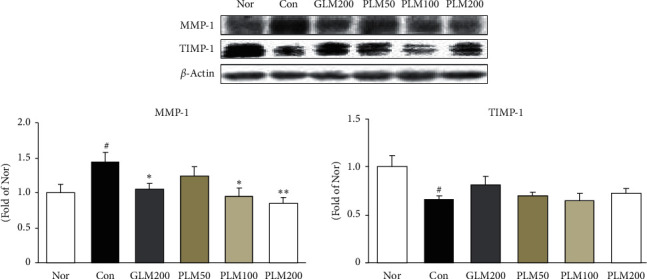
MMP-1 and TIMP-1 protein expressions in cartilage tissues. Values are the mean ± SEM (*n* = 6). Nor: normal rats; Con: MIA-induced osteoarthritis rats; GLM200: MIA-induced osteoarthritis rats administrated with green-lipped mussel 200 mg/kg body weight; PLM50: MIA-induced osteoarthritis rats administrated with PLM 50 mg/kg body weight; PLM100: MIA-induced osteoarthritis rats administrated with PLM 100 mg/kg body weight; PLM200: MIA-induced osteoarthritis rats administrated with PLM 200 mg/kg body weight. Significance: ^#^*P*< 0.05 vs. normal rat values and ^∗^*P* < 0.05, ^∗∗^*P* < 0.01 vs. MIA control rat values.

Cytokines upregulate metalloproteinase (MMPs) gene expression, and such prolytic enzymes degrade extracellular matrix, whereas TIMP-1 inhibits MMP-induced ECM degradation for the maintenance of homostasis [41]. Our results in this study revealed that PLM200 significantly suppressed the expression of MMP-1 (*P* < 0.01), otherwise elevated TIMP-1 without a significance (Figure 5).

### 3.6. Catalase and Gpx-1/2 in Cartilage Tissues

**Figure 6:**
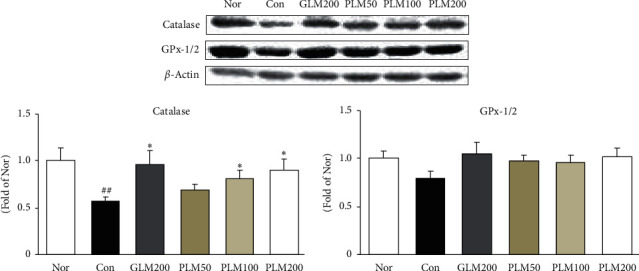
Catalase and GPx-1/2 protein expressions in cartilage tissues. Values are the mean ± SEM (*n* = 6). Nor: normal rats; Con: MIA-induced osteoarthritis rats; GLM200: MIA-induced osteoarthritis rats administrated with green-lipped mussel 200 mg/kg body weight; PLM50: MIA-induced osteoarthritis rats administrated with PLM 50 mg/kg body weight; PLM100: MIA-induced osteoarthritis rats administrated with PLM 100 mg/kg body weight; PLM200: MIA-induced osteoarthritis rats administrated with PLM 200 mg/kg body weight. Significance: ^##^*P* < 0.01 vs. normal rat values and ^∗^*P* < 0.05 vs. MIA control rat values.

The activity of ROS is balanced by enzymatic antioxidants such as catalase and GPx-1/2 [42]. Antioxidant properties could reinforce the cellular antioxidant status. In our results, MIA control rats showed decreased expressions of catalase and GPx-1/2 compared with those of normal rats; however, PLM administration effectively upregulated these levels. Herein, antioxidant enzyme catalase significantly increased compared with MIA control rats by GLM200, PLM100, and PLM200 supplementation (*P* < 0.05) (Figure 6).

## 4. Discussions

Osteoarthritis (OA) is a chronic and degenerative joint disease characterized by intra-articular inflammation and cartilage degradation [43]. Thus, inhibition of inflammation has been proposed as an effective strategy for improving the symptom or delaying the progression of OA. NSAIDs are the typical prescribed medications for treating disorders such as OA. But, the adverse effects about long-term uses of NSAIDs still existed [44]. Phytochemicals can modulate inflammatory response and treat OA [45]. Such nature-derived compounds have recently been paid attention to ideal drugs for OA due to their excellent anti-inflammatory activities and limited adverse effects [46, 47].

*Phellinus linteus* mycelium (PLM), a well-known medicinal mushroom, has been used in Asian countries for many centuries to prevent or treat diseases such as gastroenteric dysfunction, hemorrhage, diarrhea, rheumatoid arthritis, and cancers [48]. Our findings revealed that PLM inhibited inflammatory responses in MIA-induced osteoarthritis rats. To the best of our knowledge, this is regarded the first report to demonstrate the chondroprotective effect of PLM- on MIA-induced OA. Above all, hindpaw weight-bearing distribution (HWBD) is measured. HWBD, as a measure of OA progression, was measured as the difference between MIA-injected and contralateral hind limbs. The groups with GML200 and PLM administration displayed a higher HWBD compared with that of the MIA control group. Especially, PLM200 showed a significant increase. The pain of cartilage leads to decrease of HWBD, which has been proved by several studies [49, 50]. These results demonstrate a balance and relief of cartilage discomfort in the PLM-treated group.

As mentioned earlier, the degradation of cartilage results from the imbalance between catabolic activity and the mechanical stresses, and the latter is primarily controlled by ROS and MMPs. Thus, our results can be explained that the antioxidant defense of PLM decreased the cartilage damage by inhibition of the formation of ROS and MMPs. Disruption of the cartilage homeostasis leads to cartilage destruction. ROS-induced damage is significantly greater in OA cartilage. ROS in the OA process is focused in particular on NADPH oxidase, which is heme-containing transmembrane proteins and especially includes Nox4, p47^phox^, and p22^phox^. Ultimately, NADPH oxidase catalyses the transfer of the electron from NADPH and generates superoxide anion (O_2_^−^), giving rise to ROS [38, 51]. We measured protein expressions of NADPH oxidases associated with ROS production to investigate the role of PLM. Herein, excessive production of ROS damages nucleic acids, protein, lipids, and matrix components [52]. Mitochondria are essential for cellular bioenergetics and regarded as the major cellular site for ROS production [38]. Our present data indicate that treatment with PLM200 decreased NOX4, p47^phox^, and p22^phox^ production in the cartilage. H_2_O_2_ is regarded as a representative form of ROS, and accumulation of H_2_O_2_ is linked closely to the progression of OA [32]. In the previous study, H_2_O_2_-induced oxidative stress microenvironment and catalase and GPx-1/2 neutralize H_2_O_2_ to form H_2_O [52]. After MIA injection, the antioxidant enzymes including catalase and GPx-1/2 were increased than those of the MIA control group. PLM treatment showed a significant upregulation of catalase, which alleviated the degradation of ECM and protected the cartilage from oxidative stress. As the result, this suggests that the antioxidant activity of PLM may contribute to reducing pain in the MIA rat model of OA.

In addition, we examined the expression of matrix metalloproteinase (MMP)-1 and TIMP-1. The extracellular matrix is composed of proteoglycans (aggrecans) that retain water and of collagen II (95%). MMPs are a class of proteolytic enzymes involved in the occurrence, development, and progression of OA that promote the degradation of cartilage matrix components such as collagen II, which is a crucial factor in balancing the synthesis and degradation of the ECM of articular cartilage [16, 53]. Among MMPs, MMP-1 is a soluble proteinase known as collagenase-1 and degrades the extracellular matrix of cartilage and plays a main role in the occurrence, development, and progression of OA [16]. Meanwhile, activated MMP-1 is mainly regulated by tissue inhibitor of metalloproteinase (TIMP)-1 as the chief MMP regulator. Moreover, TIMP-1 is produced by connective tissue and resists against collagenase, gelatinase, and stromelysin [54, 55]. In this study, MIA control expressed an increased MMP-1 expression, and it also suggested that MMP-1 is associated with destruction of cartilage. However, PLM supplementation significantly blocked the MMP-1 upregulation of the MIA control group. These results suggest that the anticatabolic effect of PLM may be due to the inhibition of MMP-1.

Mitogen-activated protein kinases (MAPKs), which are a family of serine/threonine kinases, integrate and process various extracellular signals or cytokines [56]. The p38 MAPK corresponds to an integral component of the proinflammatory signaling pathway about external stress signals and is associated with inflammation, cell differentiation, cell growth, and cell death [56, 57]. Several studies have shown that blocking the p38 activity attenuates the transcriptional activity of the proinflammatory transcription factor NF-*κ*B without altering its DNA-binding activity. The NF-*κ*B signaling pathway is involved in the regulation of inflammatory mediators and cytokines in the progression of OA [58]. NF-*κ*B is a transcription factor localized to the cytoplasm and is rendered inactive by a constitutive interaction with the inhibitory protein I*κ*B [38]. NF-*κ*Bp65 is separated from I*κ*B and translocates to the nucleus to regulate the expression of inflammatory mediators and cytokines [59]. Many studies have demonstrated inflammatory cytokines such as IL-1*β*, TNF-*α*, and IL-6 play vital roles in OA pathogenesis [37, 51], which is intimately related to degradation of the extracellular matrix (ECM). Besides, COX is a key proinflammatory enzyme that converts arachidonic acid into prostaglandins. The excessive iNOS induces the overproduction of NO. NO generates ONOO^−^ through reacting with O_2_^−^, and this can accelerate OA [60]. Inhibition of NF-*κ*B activation is associated with the downregulation of COX-2 expression and synthesis. Moreover, higher levels of these cytokines in the initiation and progression of articular cartilage destruction have been shown to correlate with pain and physical function of patients with OA [61]. Our results suggest that suppression of NF-*κ*B activation may underlie the mechanism by which PLM inhibits the inflammatory response in cartilage tissues. That is, the elevated expressions of COX-2 and iNOS were markedly attenuated by both PLM100 and PLM200 treatments, and three cytokines such as IL-1*β*, TNF-*α*, and IL-6 significantly decreased by PLM200.

## 5. Conclusions

**Figure 7:**
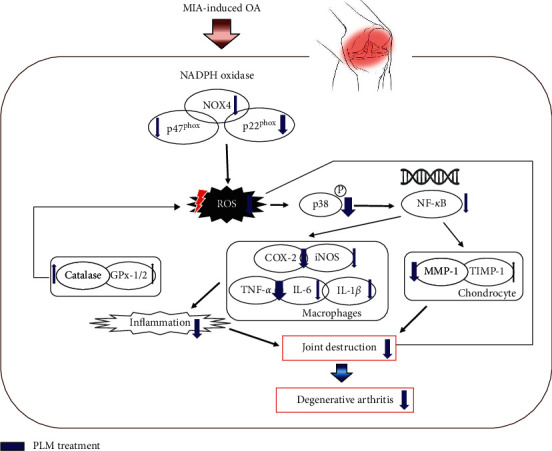
Possible mechanism of *Phellinus linteus* mycelium (PLM) in MIA-induced osteoarthritis rats.

Taken together, it suggests that PLM could inhibit excessive production of ROS and elevate anti-oxidant effect through catalase upregulation. Moreover, PLM could lead to NF-*κ*B inactivation through inhibition of p38 MAPK and it will suppress sequentially both inflammation factors and components related to degradation of cartilage matrix (Figure 7). As the result, PLM treatment may be the potential therapeutic candidate in patients with osteoarthritis.

## Figures and Tables

**Table 1:** Powder manufacturing process of *Phellinus linteus* mycelium extract.

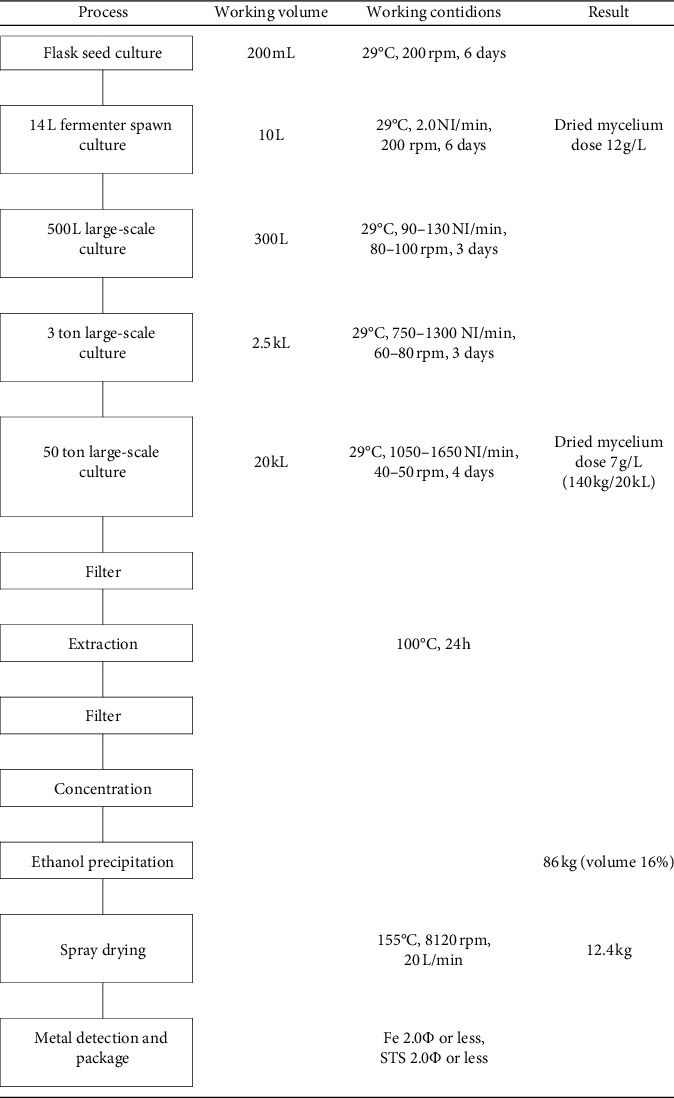

**Table 2:** General nutrition compositions of *Phellinus linteus* mycelium.

Calorie (kcal/100 g)	Carbohydrate (%)	Protein (%)	Fat (%)	Sodium (mg/100 g)	Sugar (mg/g)	Saturated fatty acid (g/100 g)	Transfat (g/100 g)	Cholesterol (mg/100 g)
346.36	68.86	15.75	0.88	71.57	128.29	0.07	0	0

## Data Availability

The data used to support the findings of this study are included within the article.
